# Lean mass development trajectories in children and adolescents and their associations with musculoskeletal health in emerging adulthood

**DOI:** 10.3389/fendo.2026.1898386

**Published:** 2026-08-14

**Authors:** M. Marques, F. Baptista, O. Rysavy, C. Pendleton, S. M. Levy, K. F. Janz

**Affiliations:** 1 CIPER, Faculdade de Motricidade Humana, Universidade de Lisboa, Lisbon, Portugal; 2 Division of Biostatistics and Computational Biology, University of Iowa, Iowa City, IA, United States; 3 Department of Epidemiology, University of Iowa, Iowa City, IA, United States; 4 Department of Health and Human Physiology, University of Iowa, Iowa City, IA, United States

**Keywords:** body composition trajectories, bone mineral density, fat mass, lean mass, MDCT, pQCT, young adults

## Abstract

This study identified longitudinal trajectories of lean mass development from childhood to young adulthood and examined their associations with bone and muscle outcomes in early adulthood. Data were obtained from 323 participants (8–23 years) in the Iowa Bone Development Study, assessed at 7 time points. Lean mass trajectories were defined using DXA-derived Z-scores for the appendicular lean mass index (aLBMI) and the appendicular lean mass to trunk fat mass ratio (aLBM/TrFM), categorized as low, average, or high. Only participants with consistent trajectories were included (n=116 for aLBMI; n=67 for aLBM/TrFM). At 23 years, bone material (DXA aBMD and pQCT vBMD), bone structure (pQCT geometry and strength), and muscle composition (pQCT muscle area and density) were assessed. Linear regression used the average trajectory as a reference and was adjusted for sex and height. A low aLBMI trajectory was associated with lower aBMD at the whole body less head (WBLH), femoral neck, and trochanter (−8% to −11%) and reduced tibial structural strength (−23% to −34%), with no differences at the radius. Higher aLBMI trajectories were associated with higher aBMD (up to +17%), greater bone strength in the tibia and radius (+22 to 45%), and larger muscle area (+32%). For aLBM/TrFM, only WBLH aBMD differed, with the high group showing ~5% higher values, along with lower BMI and higher muscle density. These findings suggest that consistent longitudinal patterns of lean mass development are associated with bone and muscle outcomes in early adulthood, with aLBMI more closely related to bone structure and muscle area, while a favorable muscle-to-fat ratio may reflect better muscle quality and whole-body bone status.

## 1 Introduction

Traditional anthropometric indicators such as body mass index (BMI) and waist circumference (WC) have important limitations for assessing health risk, particularly during growth (1). BMI does not distinguish between lean and fat mass, while WC, although related to visceral adiposity, provides only an indirect estimate of body composition (2). These limitations underscore the importance of directly assessing body composition during childhood and adolescence, when body composition and musculoskeletal tissues undergo rapid development (3).

Low muscle mass and the combination of low muscle mass with excess adiposity have been associated with adverse metabolic, cardiovascular, functional, and skeletal outcomes (4–6). During growth, skeletal muscle contributes to movement, glucose disposal, protein turnover, and mechanical loading, whereas excess adiposity may contribute to insulin resistance, inflammation, and impaired metabolic function (7). This muscle-fat interaction also extends to the skeleton, as bone development depends on both the mechanical loading derived from lean mass and the systemic effects of adipose tissue (8). However, the longitudinal development of lean mass (as a surrogate for muscle) and fat mass, and their associations with bone and muscle outcomes in early adulthood, remain incompletely characterized.

Bone health can be described through two complementary domains. Material properties reflect the intrinsic quality of the tissue, such as mineral density (9), whereas structural properties encompass the bone’s architecture, including geometry, mass distribution, and microarchitecture, which determine its ability to withstand bending, torsion, and compression (10–12). Because these domains may respond differently to growth and mechanical loading, assessing both is important for understanding musculoskeletal development (13).

Although boys and girls follow distinct biological patterns of body composition development, particularly the greater lean mass accrual in boys during puberty, driven by androgenic stimulation, which enhances bone strength (14), the mechanisms linking muscle, fat, and bone operate across both sexes. Understanding these developmental patterns may help clarify early-life contributors to later osteoporosis and sarcopenia risk (15), particularly in increasing pediatric obesity and physical inactivity (16).

Therefore, this study aimed to characterize longitudinal trajectories of lean mass from childhood to young adulthood using the appendicular lean mass index (aLBMI) and the ratio of appendicular lean mass to trunk fat mass (aLBM/TrFM) and to examine their associations with musculoskeletal outcomes in emerging adulthood, including muscle area and composition, bone material properties, and bone structural properties (microarchitecture and strength). Using data from the Iowa Bone Development Study (IBDS), we sought to identify body composition profiles associated with more and less favorable of musculoskeletal development.

## 2 Methods

### 2.1 Sample design and data collection

The IBDS is a long-term cohort study examining how bone and body composition develop from childhood through young adulthood (17–19). Participants were selected from the Iowa Fluoride Study birth cohort, which recruited 1,882 families from eight Iowa hospital maternity wards between 1992 and 19957 (20). Enrollment in the IBDS occurred mostly from 1998 to 2000, when the children were about 5 years old, using a rolling admissions process that also allowed some participants to join at later follow-up waves. Participants underwent assessments approximately every two to three years. This analysis includes data from visits conducted between ages 8 and 23 years (2000-2018), with the age 23 visit corresponding to the young-adult follow-up wave and the final available assessment for the present analysis. Health questionnaires documented medical conditions that can affect bone development (e.g., scoliosis, kyphosis, diabetes, arthritis, asthma). During middle childhood, questionnaires were completed by parents; during late childhood, by children with parental assistance; and during adolescence and young adulthood, by participants independently. Bone outcomes at the proximal femur were slightly lower among participants with current asthma; however, after adjustment for body size and sex, these differences were not statistically significant.

The study enrolled 323 male and female participants. Trajectories were defined using Z-scores for appendicular lean mass index relative to height (aLBMI) and appendicular lean mass relative to trunk fat mass (aLBM/TrFM): low (≤ -1), average (−1 to +1), and high (> +1). For this analysis, only individuals who consistently stayed within the same mass trajectory throughout follow-up were included. This criterion resulted in final analytic samples of 116 participants for the aLBMI trajectories and 67 for the aLBM/TrFM trajectories. The study protocol received approval from the University of Iowa Institutional Review Board for Human Subjects, and informed consent or assent was obtained from all participants, along with informed consent from parents when children were under age 18.

### 2.2 Dual-energy X-ray absorptiometry

Dual-energy X-ray absorptiometry (DXA) scans were performed at age 23 using a Hologic QDR-4500A densitometer (Delphi upgrade, software v12.3, fan-beam mode). DXA assessed both body composition (including appendicular lean mass and trunk fat mass, used to calculate aLBMI and aLBM/TrFM Z-scores) and areal bone mineral density (aBMD). The aLBMI was standardized (Z-scores) based on reference ranges for body composition indices (21). The ALST/TrFM was standardized using the sample as a reference. The aBMD measurements included the whole body minus the head (WBLH), arms and legs (used to determine the arms-to-legs aBMD ratio), and proximal femur (femoral neck and trochanter). Quality assurance involved daily spine phantom scans, and all measurements were taken by trained technicians, with a coefficient of variation less than 1% for bone mineral content based on routine quality-control phantom scans.

### 2.3 Peripheral quantitative computed tomography

Peripheral quantitative computed tomography (pQCT, Stratec XCT 2000/3000, software version 6.2) was used to evaluate volumetric bone density, bone strength indices and muscle composition at the standardized sites of the non-dominant radius and tibia (Stratec XCT 2000/3000, software version 6.2). Limb length was measured from anatomical landmarks (radius: radial styloid to olecranon; tibia: medial malleolus to tibial plateau) to standardize scan locations. Slice thickness was 2.2 mm, with a voxel size of 0.4 mm^2^. All scans were visually inspected for motion artefacts, and daily scans of manufacturer-provided hydroxyapatite phantoms were performed to ensure quality assurance.

Measurements at the 4% metaphyseal site of the radius and tibia included trabecular volumetric BMD (TrBMD) and trabecular bone strength index (BSI Tr). At the 20% radius and 38% tibia diaphyseal sites, we assessed cortical volumetric BMD (CoBMD) and calculated bone strength indices: polar moment of inertia, moment of resistance, and cortical bone strength index (BSI Co). Muscle composition (muscle cross-sectional area and muscle density) was evaluated at the 66% tibia site.

### 2.4 Multi-detector computed tomography

High-resolution multi-detector computed tomography (MDCT) was performed to evaluate cortical and trabecular bone microstructure at the distal tibia. Scans were acquired using a 128-slice SOMATOM Definition Flash scanner (Siemens, Munich, Germany) following the Iowa Bone Development Study protocol (22). The left tibia was scanned unless contraindicated by prior injury. Laser positioning and anterior-posterior scout imaging ensured alignment of the tibial axis with the scanner’s isocenter. Scans were obtained in ultra-high-resolution mode (120 kV, 200 mAs, pitch 1.0, collimation 16 × 0.6 mm), reconstructed at 400-µm slice thickness and 200-µm spacing, and resampled to an isotropic voxel size of 150 µm. A tissue-characterization phantom (Gammex RMI 467) was scanned to convert Hounsfield units to volumetric BMD (mg/cm³). After motion-quality screening, validated automated algorithms were used to segment cortical and trabecular compartments and calculate microstructural indices, including trabecular volumetric BMD, trabecular thickness, cortical thickness, and cortical porosity.

### 2.5 Additional measurements

Height (cm) and weight (kg) were measured by trained research nurses using standardized equipment, a Healthometer scale and a Harpenden stadiometer, with regular accuracy checks.

### 2.6 Statistical analysis

Descriptive statistics are presented as estimated marginal means with standard errors (EMM ± SE). Between-group differences in bone and muscle outcomes at age 23 years were examined using analysis of covariance (ANCOVA). DXA-derived variables were adjusted for sex and height, whereas pQCT- and MDCT-derived variables were adjusted for sex only, as these techniques provide volumetric and structural measures that are less dependent on body size. *Post-hoc* pairwise comparisons used a Sidak correction for multiple testing.

Outcomes included DXA-based areal BMD (WBLH, femoral neck, trochanter, arms-to-legs ratio), pQCT-based volumetric density, geometry, and strength indices (trabecular and cortical vBMD, bone strength index, polar moment of inertia, moment of resistance), muscle area and density, and MDCT-derived cortical and trabecular microstructure (cortical porosity and trabecular indices). Complementary bivariate linear regression analyses were conducted using the average trajectory as the reference category. Only variables that showed significant between-group differences in the ANCOVA were included in these regression analyses. Models were run separately for each outcome (one predictor at a time), and no interaction terms were tested. The average trajectory group served as the reference category because it represents the normative pattern of lean mass development (Z-score between −1 and 1), providing a physiologically meaningful and statistically stable comparator against which deficits (low trajectory) or advantages (high trajectory) can be evaluated.

Model assumptions (normality, homoscedasticity, linearity) were checked through visual inspection of diagnostic plots, and variance homogeneity was tested with Levene’s test. Statistical significance was established at p < 0.05, and analyses were conducted using IBM SPSS Statistics 29.0 (IBM Corp., Armonk, NY, USA).

## 3 Results

**Figure 1:**
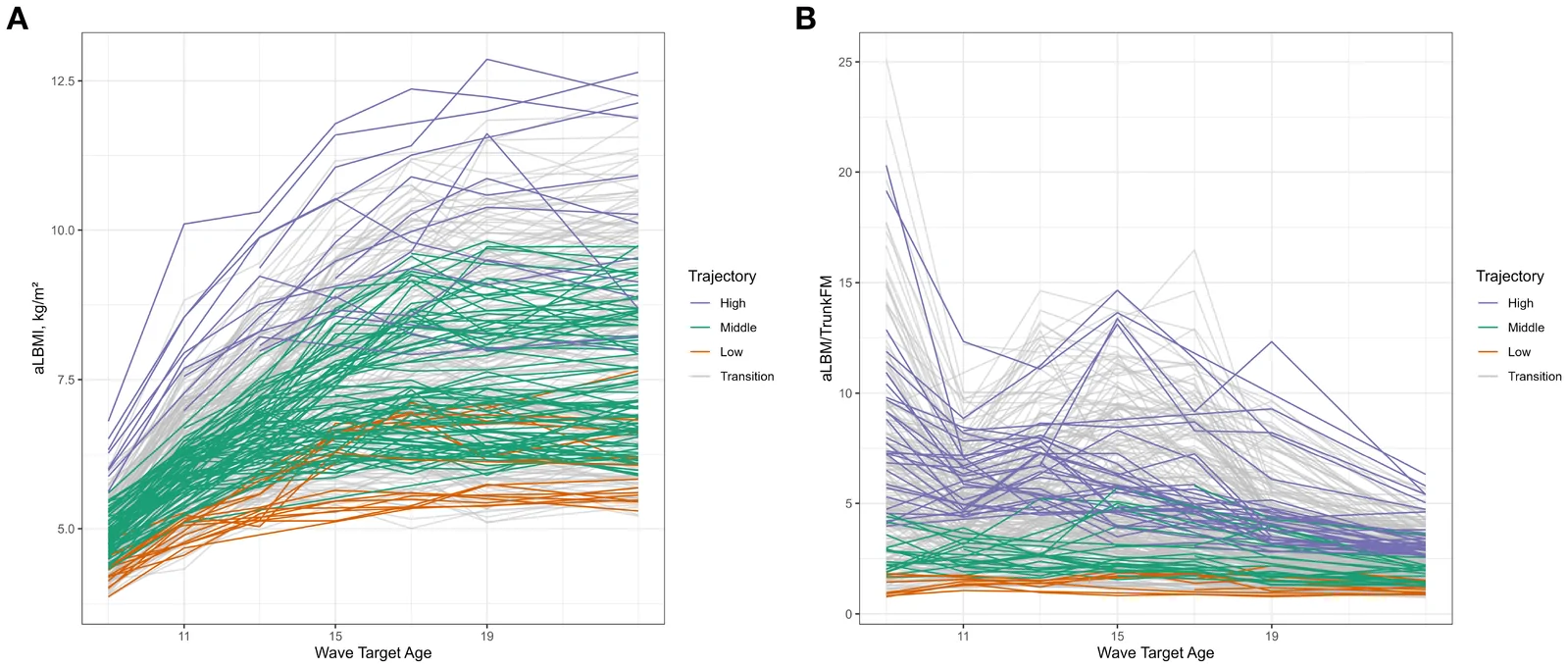
Trajectory groups of lean mass defined by Z-score consistency from age 8 to 23 for aLBMI **(A)** and aLBM/TrFM **(B)**.

**Figure 1** shows the paths of different participants for aLBMI and aLBM/TrFM. The trajectories reveal that most participants did not stay in the same category, low, average, or high, throughout the observation period. Therefore, this analysis focuses on participants with steady trajectories, defined as those who remained within the same category across repeated assessments from childhood to young adulthood. A body composition risk (Z score < -1) was maintained in 5% of participants when using the aLBMI index as a reference, or in 3% when using the aLBM/TrFM as a reference.

**Table 1** presents descriptive statistics (EMMs ± SE) for bone and muscle variables by trajectory group, derived from ANCOVA models adjusted for sex (and height for DXA-BMD outcomes). Comparisons among the low, average, and high trajectories reveal significant differences in body composition, bone mineral density, and bone strength parameters, outlining each group’s overall profile. Compared with the average trajectory group, the low trajectory group in the aLBMI showed significantly lower body mass, BMI, and aBMD at WBLH, arms, legs, femoral neck, and trochanter, while the high trajectory group showed significantly higher values for these regions, except the arms/legs ratio. Similar trends were observed for parameters derived from pQCT: the low aLBMI group displayed lower tibial and radial bone strength indices, including polar moment of inertia, moment of resistance, and BSI, while the high trajectory group exhibited higher values for these variables. Measures of bone microstructure (MDCT) revealed slightly higher cortical porosity in the high group, with no significant differences in trabecular thickness or separation. Regarding muscle parameters, muscle area followed a similar pattern, smaller in the low group and larger in the high group, while muscle density was consistent across trajectories.

**Table 1:** Characteristics of participants according to appendicular lean body mass index and the ratio of appendicular lean body mass to trunk fat mass (estimated marginal mean ± standard error).

		aLBMI				aLBM/TrFM			
Variable	Method	Low Z-score ≤ -1 n=16	Average -1 < Z-score ≤ 1 n=86	High z-score > 1 n=14	p	Low Z-score ≤ -1 n=9	Average -1 < Z-score ≤ 1 n= 24	High z-score > 1 n= 34	p
Age, y		23.6 ± 0.2	23.4 ± 0.1	23.8 ± 0.2	0.368	23.5 ± 0.2	23.5 ± 0.1	23.5 ± 0.1	0.515
Height, cm		170.2 ± 1.9	172.8 ± 0.8	174.5 ± 1.9	0.053	170.1 ± 2.9	173.9 ± 1.4	172.2 ± 1.3	0.292
Body mass, kg		60.6 ± 3.4	75.8 ± 1.4	106.6 ± 3.5	<0.001	110.5 ± 5.4	88.2 ± 2.6	64.6 ± 2.3	0.067
BMI, kg/m^2^		20.9 ± 1.0	25.2 ± 0.4	35.0 ± 1.1	0.006	37.8 ± 1.5	29.0 ± 0.7	21.5 ± 0.6	0.009
Bone density									
WBLH aBMD, g/cm^2^	DXA	1.001 ± 0.020	1.093 ± 0.011	1.169 ± 0.020	<0.001	1.064 ± 0.029	1.053 ± 0.017	1.104 ± 0.140	0.025
Arms aBMD, g/cm^2^	DXA	1.547 ± 0.030	1.643 ± 0.015	1.767 ± 0.029	0.003	1.752 ± 0.251	1.652 ± 0.249	1.608 ± 0.198	0.122
Legs aBMD, g/cm^2^	DXA	2.316 ± 0.048	2.531 ± 0.025	2.750 ± 0.048	<0.001	2.736 ± 0.344	2.538 ± 0.335	2.470 ± 0.288	0.089
Arms/legs aBMD	DXA	0.670 ± 0.001	0.650 ± 0.005	0.641 ± 0.010	<0.001	0.623 ± 0.014	0.651 ± 0.008	0.657 ± 0.007	0.092
Fem Neck aBMD, g/cm^2^	DXA	0.833 ± 0.148	0.937 ± 0.139	1.100 ± 0.114	<0.001	1.013 ± 0.049	0.923 ± 0.029	0.947 ± 0.024	0.297
Trochanter aBMD, g/cm^2^	DXA	0.715 ± 0.128	0.798 ± 0.125	0.914 ± 0.124	<0.001	0.832 ± 0.041	0.786 ± 0.024	0.803 ± 0.020	0.609
Radius TrBMD, mg/cm^3^	pQCT	204 ± 9	204 ± 4	233 ± 9	0.985	193 ± 13	199 ± 8	201 ± 6	0.860
Radius CoBMD mg/cm^3^	pQCT	673 ± 12	664 ± 5	679 ± 13	0.296	666 ± 17	670 ± 10	660 ± 9	0.772
Tibia TrBMD, mg/cm^3^	pQCT	250 ± 9	259 ± 3	275 ± 9	0.109	245 ± 13	249 ± 8	258 ± 7	0.574
Tibia CoBMD, mg/cm^3^	pQCT	653 ± 12	659 ± 5	686 ± 13	0.091	670 ± 20	604 ± 12	653 ± 10	0.644
Micro structure (Tibia)									
Tb thickness, µm	MDCT	133 ± 1	134 ± 1	141 ± 6	0.159	129 ± 5	128 ± 3	136 ± 2	0.076
Tb separation, µm	MDCT	453 ± 29	404 ± 13	349 ± 32	0.062	488 ± 67	434 ± 29	411 ± 34	0.608
Co porosity	MDCT	0.175 ± 0.003	0.181 ± 0.001	0.194 ± 0.003	<0.001	0.191± 0.004	0.185± 0.002	0.181± 0.002	0.086
Bone strength									
Radius polar moment of inertia Co, mm^4^	pQCT	1491 ± 139	2180 ± 60	2841 ± 146	<0.001	2795 ± 262	2570 ± 164	2409 ± 137	0.076
Radius moment of resistance, mm^3^	pQCT	211 ± 13	271 ± 5	331 ± 13	<0.001	318 ± 25	302 ± 14	287 ± 12	0.194
Radius BSI Tr, mg^2^/mm^4^	pQCT	7.0 ± 0.9	8.0 ± 0.4	11.6 ± 0.9	<0.001	8.4 ± 1.4	8.0 ± 0.9	7.9 ±0.7	0.940
Radius BSI Co mg^2^/mm^4^	pQCT	48 ± 3	53 ± 1	65 ± 3	<0.001	50 ± 4	54 ± 2	52 ± 2	0.695
Tibia polar moment of inertia Co, mm^4^	pQCT	19442 ± 1796	29335 ± 774	39029 ± 2011	<0.001	40037 ± 3117	33353 ± 1949	31804 ± 1630	0.005
Tibia moment of resistance, mm^3^	pQCT	1411 ± 87	1835 ± 37	2247 ± 92	<0.001	2264 ± 139	2000 ± 87	1910 ± 73	0.021
Tibia BSI Tr, mg^2^/mm^4^	pQCT	36 ± 3	47 ± 1	58 ± 4	0.003	42 ± 5	43 ± 3	46 ± 2	0.555
Tibia BSI Co, mg^2^/mm^4^	pQCT	81 ± 5	99 ± 2	126 ± 6	<0.001	103 ± 9	101 ± 5	94 ± 3	0.502
Muscle									
Muscle area, cm^3^	pQCT	5739 ± 229	7349 ± 97	9739 ± 239	<0.001	8854 ± 385	7930 ± 227	7009 ± 227	<0.001
Muscle density, mg/cm^3^	pQCT	79.6 ± 0.4	79.8 ± 0.2	79.1 ± 0.4	0.346	77.3 ± 0.3	79.5 ± 0.2	80.3 ± 0.2	<0.001

BMI, body mass index; aLBM, appendicular lean body mass; aLBMI, appendicular lean body mass index; TrFM, trunk fat mass; WBLH, whole body less head; BMD, bone mineral density; Fem, femoral; Tr, trabecular; Co, cortical; BSI, bone strength index; DXA, dual-energy X-ray absorptiometry; pQCT, peripheral quantitative computed tomography; MDCT, multidetector computed tomography.

Values are estimated marginal means (EMMs) derived from ANCOVA models adjusted for sex (and height for DXA-BMD outcomes), with Sidak-corrected post-hoc comparisons.

P-values correspond to the overall trajectory group effect from ANCOVA F-tests..

For aLBM/TrFM trajectories, participants in the low group had higher body mass and BMI but lower WBLH BMD, whereas those in the high trajectory group showed lower body mass and BMI along with higher WBLH aBMD. This high aLBM/TrFM group also demonstrated greater muscle density and smaller muscle area, indicating a leaner composition. Additionally, tibial bone strength indices varied significantly across aLBM/TrFM trajectories: the low group, characterized by higher body mass, showed higher values for tibial polar moment of inertia and moment of resistance, while these values were lower in the average and high groups.

**Table 2** summarizes the results of the linear regression analyses, adjusted for sex and height (DXA) or sex only (pQCT/MDCT). These models assess the differences among trajectory groups, using the average trajectory as the reference. Comparisons were made separately for the low and high trajectories, relative to the average group, to assess the strength of the associations. Since these pairwise models compare each trajectory only with the reference, instead of testing all three groups at once, some differences seen in **Table 1** were reduced or no longer significant.

**Table 2:** Linear regression models examining the associations of appendicular lean body mass index (aLBMI) and lean body mass/trunk fat mass ratio (aLBM/TrFM) trajectory groups with bone and muscle outcomes.

	aLBMI			aLBM/TrFM		
	B	95% CI	p	B	95% CI	p
WBLH BMD						
Low Trajectory	-0.097	-0.014; -0.570	0.001	–	–	–
High Trajectory	0.039	0.019; -0.060	< 0.001	0.025	0.002 - 0.048	0.031
Arms/legs BMD						
Low Trajectory	–	–	–	–	–	–
High Trajectory	–	–	–	–	–	–
Fem neck BMD						
Low Trajectory	-0.117	0.185; -0.048	0.001	–	–	–
High Trajectory	0.069	0.035; - 0.103	< 0.001	–	–	–
Troch BMD						
Low Trajectory	-0.096	-0.156; -0.036	0.002	–	–	–
High Trajectory	0.047	0.018; - 0.077	0.002	–	–	–
Height						
Low Trajectory	–	–	–	–	–	–
High Trajectory	–	–	–	–	–	–
Co porosity						
Low Trajectory	–	–	–	–	–	–
High Trajectory	–	–	–	–	–	–
Radius polar moment of inertia Co						
Low Trajectory	–	–	–	–	–	–
High Trajectory	427	171; 683	0.001	–	–	–
Radius moment of resistance						
Low Trajectory	–	–	–	–	–	–
High Trajectory	39	16; 62	0.001	–	–	–
Radius BSI Tr						
Low Trajectory	–	–	–	–	–	–
High Trajectory	2.2	0.9; 3.4	<0.001	–	–	–
Radius BSI Co						
Low Trajectory	–	–	–	–	–	–
High Trajectory	6.8	2.5; 11.2	0.003	–	–	–
Tibia moment of resistance						
Low Trajectory	−306	−556; −56	0.017	–	–	–
High Trajectory	248	116; 380	<0.001	–	–	–
Tibia polar moment of inertia Co						
Low Trajectory	−7218	−12630; −1 808	<0.001	12256	1023; 23489	0.034
High Trajectory	5821	2879; 8763	<0.001	–	–	–
Tibia BSI Tr						
Low Trajectory	–	–	–	–	–	–
High Trajectory	7	2; 12	0.006	–	–	–
Tibia BSI Co						
Low Trajectory	–	–	–	–	–	–
High Trajectory	16	8; 25	<0.001	–	–	–
Muscle area, cm^3^						
Low Trajectory	−451	−691; −211	<0.001	–	–	–
High Trajectory	426	284; 568	<0.001	−229	−404; −54	0.011
Muscle density, mg/cm^3^						
Low Trajectory	–	–	–	−2.2	−3.1; −1.4	< 0.001
High Trajectory	–	–	–	0.5	0.2; 0.7	< 0.001

aLBM, appendicular lean body mass; aLBMI, appendicular lean body mass index; TrFM, trunk fat mass; WBLH, whole body less head; BMD, bone mineral density; Troch, trochanter; Co, cortical; Tr, trabecular; BSI, bone strength index. Regression models adjusted for sex and height, except for the height model, which was adjusted only for sex. Only variables showing significant group effects in the ANCOVA (**Table 1**) were included in these regression models. The symbol ‘–’ indicates no significant difference between trajectory groups compared with the average group.

For aLBMI, participants in the low trajectory exhibited lower WBLH aBMD, while those in the high trajectory showed higher values. Similar patterns appeared at regional bone sites: the femoral neck and trochanter. Regarding bone mechanical competence assessed by pQCT, significant differences were also found across aLBMI trajectories. High aLBMI trajectories were associated with higher radius polar moment of inertia, moment of resistance, and trabecular radius bone strength index. At the tibial site, low trajectories showed lower mechanical resistance, while high trajectories showed enhanced values. Similar patterns were also observed for muscle composition.

Participants with low aLBMI trajectories had lower muscle area, whereas those with high aLBMI trajectories had higher muscle area. Across aLBM/TrFM trajectories, muscle area tended to be smaller, while muscle density was higher from low to high groups, indicating a more favorable muscle composition despite smaller size.

**Figure 2:**
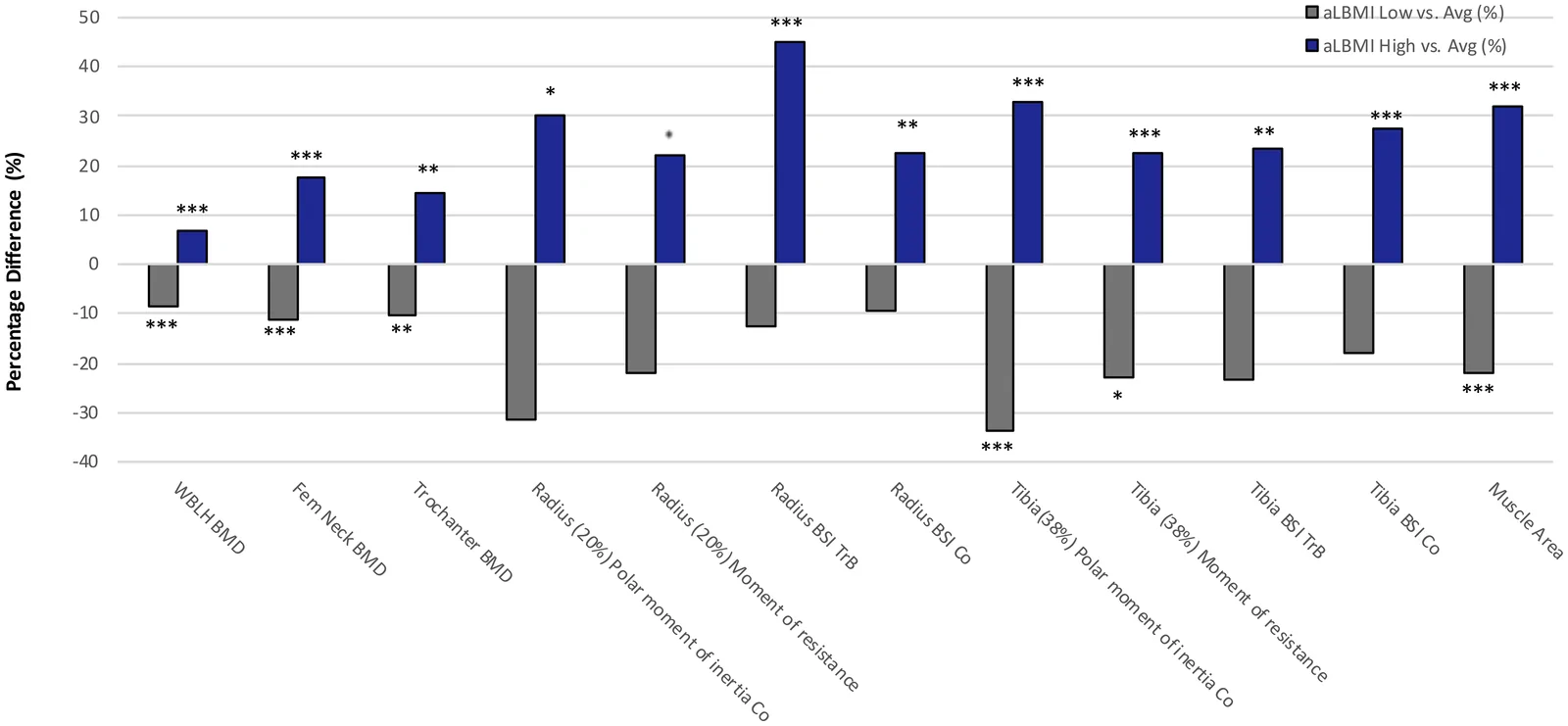
Percentage differences in bone and muscle outcomes for the low and high aLBMI trajectory groups relative to the average (Avg) group, represented by the zero line. DXA-derived variables were adjusted for sex and height; pQCT- and MDCT-derived variables were adjusted for sex only. aLBM, appendicular lean body mass; aLBMI, appendicular lean body mass index; WBLH, whole body less head; BMD, bone mineral density; Fem, femoral; Tr, trabecular; Co, cortical; BSI, bone strength index. *p < 0.05; **p < 0.01; ***p < 0.001.

In the aLBMI trajectories, bone and muscle variables showed a clear gradient relative to the average group (**Figure 2**). Participants in the high aLBMI trajectory displayed higher values for WBLH BMD, femoral neck BMD, and trochanter BMD (approximately +7% to +17%), as well as higher indices of bone geometry and strength, including the polar moment of inertia, moment of resistance, and bone strength index (BSI) at both the radius and tibia. The muscle area was also greater (+32%) in this group. Conversely, the low trajectory group showed lower values for BMD (about −8% to −11%), bone strength parameters at the tibia, and muscle area (−22%). Muscle density (**Table 2**) remained essentially unchanged (<1%).

**Figure 3:**
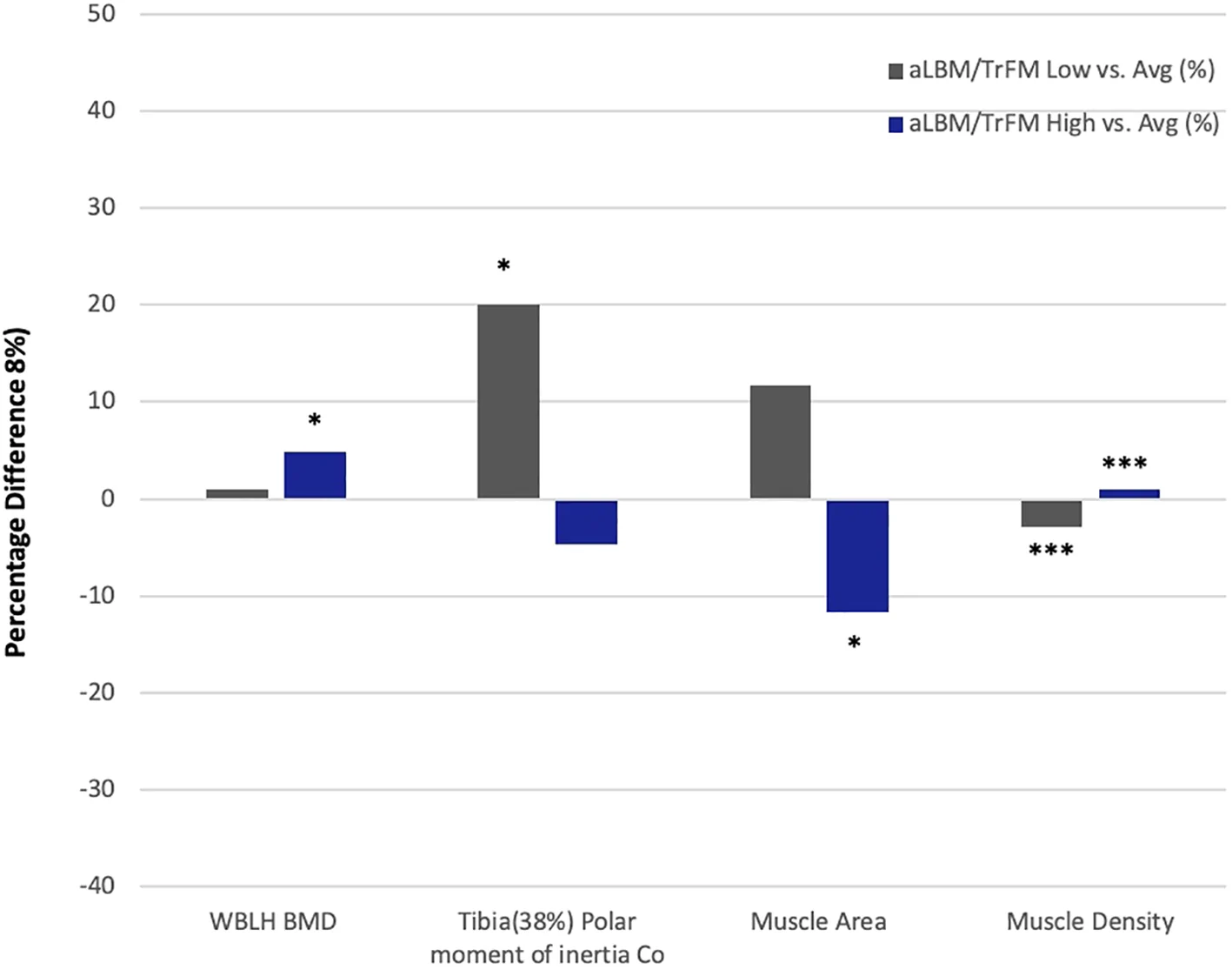
Percentage differences in bone and muscle outcomes between the low- and high-aLBM/TrFM trajectory groups and the average (Avg) group. DXA-derived variables were adjusted for sex and height; pQCT- and MDCT-derived variables were adjusted for sex only. aLBM, appendicular lean body mass; TrFM, trunk fat mass; Avg, average; WBLH, whole body less head; BMD, bone mineral density; Co, cortical. *p < 0.05; **p < 0.01; ***p < 0.001..

In the aLBM/TrFM trajectories, differences in bone variables were modest (generally within ±10%), including BMD and bone strength indices (**Figure 3**). Muscle variables showed an opposite pattern: the high aLBM/TrFM group had a smaller muscle area (−12%) but slightly higher muscle density (+1%), whereas the low group exhibited a larger muscle area (+12%) and lower muscle density (−3%).

## 4 Discussion

This study examined whether consistent longitudinal patterns of lean mass development from childhood to young adulthood were associated with skeletal outcomes, mechanical competence, and muscle composition at 23 years in a healthy cohort. Persistently low aLBMI trajectories were associated with lower aBMD across multiple skeletal regions (WBLH, femoral neck, and trochanter) and with diminished bone strength indices assessed by pQCT. In contrast, participants with high aLBMI trajectories exhibited higher bone mineralization and mechanical competence, emphasizing the critical role of lean mass accrual in skeletal development.

These findings support the concept of the “muscle-bone unit,” where bone adapts in response to mechanical and biochemical stimuli generated by muscle (6). Lower lean mass may be associated with reduced mechanical loading and osteogenic stimulation, whereas higher lean mass may reflect greater exposure to strain-related forces and myokine signaling (23). The differences in aBMD observed across aLBMI trajectories highlight the sensitivity of weight-bearing bones to muscle-derived forces, consistent with previous evidence that reduced muscle mass during growth is associated with lower bone strength and a higher risk of fractures in adulthood (24, 25).

Despite higher areal BMD, individuals with a high aLBMI trajectory did not show a corresponding advantage in volumetric BMD or microstructure. Greater cortical porosity among these individuals suggests that the associations with higher lean mass were primarily structural, reflected in bone geometry (e.g., increased cross-sectional area and moments of inertia) rather than in material properties, such as tissue mineral density. This supports mechanical models indicating that bone strength depends more on geometry than on volumetric mineralization when muscle forces are elevated (8).

Evidence indicates that, during this period, the structural response dominates, mainly driven by muscle forces associated with increasing lean mass, while material changes serve a secondary role, fine-tuning bone quality as growth slows (26–28). This difference may help explain why greater lean mass primarily supports structural, rather than material, bone advantages during development (29).

Comparisons between aLBMI and aLBM/TrFM trajectories provide additional insight into muscle–bone interactions. While aLBMI trajectories showed strong associations with bone outcomes, aLBM/TrFM trajectories were more closely associated with muscle characteristics. Participants with higher aLBM/TrFM had smaller muscle area but greater muscle density, suggesting reduced fat infiltration and more compact muscle tissue. Although major bone advantages did not parallel these differences, these findings suggest that a favorable muscle-to-fat ratio may be associated with better muscle composition, which may indirectly support bone through improved metabolic efficiency (30, 31). Conversely, excess adiposity may impair bone through inflammatory cytokines and reduced mechanical efficiency (32).

Together, these results highlight the complementary roles of muscle quantity and composition in skeletal health. Greater lean mass development was associated with more favorable bone geometry and mechanical strength, while a favorable muscle-to-fat ratio seems to compensate for lower total mass by maintaining muscle density and function. Preventing persistently low lean mass in children, especially those with low body weight or delayed growth, may be relevant for supporting optimal bone development. Early detection of individuals with low lean mass trends could allow for targeted interventions to enhance both muscle and bone growth, such as resistance training, adequate protein intake, and balanced nutrition.

Only a small proportion of participants maintained persistently low lean mass trajectories (5% for aLBMI and 3% for aLBM/TrFM), but these individuals may represent a vulnerable group with elevated risk of early-onset sarcopenia and reduced peak bone mass. Since peak bone mass achieved by early adulthood determines up to 60% of future fracture risk (15), these findings underscore the relevance of strategies that promote both muscle quantity and quality during growth.

The main strengths of this study include its 15-year longitudinal design, the integration of DXA, pQCT, and MDCT imaging, and the trajectory-based approach that captures long-term developmental patterns. By analyzing individuals with consistent body composition profiles, transient fluctuations are minimized, allowing clearer associations between muscle and bone traits to emerge. The combined use of lean mass (aLBMI) and muscle-to-fat ratio (aLBM/TrFM) provides complementary insights into musculoskeletal development.

Limitations include modest sample sizes within trajectory groups, potential residual confounding from physical activity, diet, or hormonal factors, and limited geographic and racial/ethnic diversity, which restricts generalizability. In addition, sex-stratified analyses were not performed due to the relatively small number of participants in each lean mass trajectory group, which would have limited the power to detect sex-specific associations. Nevertheless, all regression models were adjusted for sex. Given known sex differences in lean mass accrual, maturation, and bone development, future studies with larger samples should examine whether the associations between lean mass development patterns and adult musculoskeletal outcomes differ between males and females. The average trajectory group was used as the reference category in regression models because it reflects the normative developmental pattern (Z-score between −1 and 1), providing a stable and physiologically meaningful comparator for identifying deviations in muscle and bone development. However, this approach has limitations. For the aLBM/TrFM trajectories, Z-scores were derived from the sample distribution, including participants with overweight or obesity. As such, the ‘average’ category may partly reflect a population with elevated adiposity, potentially influencing the interpretability of comparisons against low and high trajectories. Future studies should consider external reference standards or larger samples to establish more robust normative curves.

## 5 Conclusions

Greater lean mass development was associated with structural rather than material bone adaptations, with similar muscle density across groups. A favorable muscle-to-fat ratio may offset lower total mass, highlighting the complementary roles of muscle quantity and composition in bone health. Interventions during growth that promote lean mass development while enhancing muscle quality may help to optimize long-term musculoskeletal integrity.

## Data Availability

The datasets presented in this article are not readily available because The Iowa Bone Development Study data contain identifiable health information and are governed by institutional data use agreements. For this reason, the datasets generated and analyzed during the current study are not publicly available. De-identified data may be made available from the Iowa Bone Development Study investigators upon reasonable request and subject to approval by the University of Iowa Institutional Review Board and the study steering committee. Requests to access the datasets should be directed to steven-levy@uiowa.edu.
